# An Innovative Method of Converting Ferrous Mill Scale Wastes into Superparamagnetic Nanoadsorbents for Water Decontamination

**DOI:** 10.3390/ma14102539

**Published:** 2021-05-13

**Authors:** Andra Mihaela Predescu, Ecaterina Matei, Andrei Constantin Berbecaru, Maria Râpă, Mirela Gabriela Sohaciu, Cristian Predescu, Ruxandra Vidu

**Affiliations:** 1 Faculty of Materials Sciences and Engineering, University Politehnica of Bucharest, 060042 Bucharest, Romania; andra.predescu@upb.ro (A.M.P.); maria.rapa@upb.ro (M.R.); mirela.sohaciu@upb.ro (M.G.S.); cristian.predescu@upb.ro (C.P.); rvidu@ucdavis.edu (R.V.); 2 Department of Electrical and Computer Engineering, University of California Davis, One Shields Avenue, Davis, CA 95616, USA

**Keywords:** innovative nanomaterials, synthesis, characterization, mill scale, water decontamination

## Abstract

The need to recycle and develop nanomaterials from waste, and use them in environmental applications has become increasingly imperative in recent decades. A new method to convert the mill scale, a waste of the steel industry that contains large quantity of iron and low impurities into a nanoadsorbent that has the necessary properties to be used for water purification is presented. The mill scale waste was used as raw material for iron oxide nanopowder. A thorough characterization was performed in each stage of the conversion process from the mill scale powder to magnetic nanopowder including XRD (X-ray diffraction), SEM (scanning electron microscopy), TEM (transmission electron microscopy), BET (Brunauer, Emmett and Teller) and magnetization properties. Iron oxide nanoparticles were approximately 5–6 nm with high specific surface area and good magnetic properties. These are the necessary properties that a magnetic nanopowder must have in order to be used as nanoadsorbents in the heavy metal removal from waters. The iron oxide nanoparticles were evaluated as adsorbents for the removal of Cu, Cd and Ni ions.

## 1. Introduction

The management of wastes resulting from the hot metal and steel industry is a critical issue due to strictly imposed environmental regulations. The waste management caused by steel industry gained major consideration as a result of these new environmental regulations. Mill scale (MS) is an unavoidable waste in steel production because MS is the result of the surface oxidation of steel during thermal and plastic deformation processes. MS is regularly washed away from the steel surfaces and removed as powders consisting of agglomerated particles of various sizes. Because of the repeated oxidation process during steel reheating, MS is mainly composed of iron oxides (>95%), metal oxides, carbon and oil [1].

Handling this type of waste can be expensive and most mineral processing plants are unable to recover the entire iron content [2]. For this reason, the reuse of this waste in the form of nanomaterials could be an economically and technically feasible method. Moreover, some of the steel-works companies dump mill scale wastes in landfills. This is a real threat to the environmental because the heavy metals from MS leach directly into the soil and groundwater [3]. In a world that is increasingly facing environmental pollution problems, it is crucial to find eco-friendly solutions with economic benefits in order to resolve them. Recycling and conversion of various ferrous wastes into smart materials can be the key for an enhanced state of environment. The continuing need for uncontaminated landfills highlights and strengthens the demand for more effective alternatives for large scale MS use.

The literature indicates the wastes resulting from finishing/cleaning operations of the finished products from rolling mills as iron source [4]. This type of waste amounts to about 35–40 kg/t rolled product, which can be used as precursor in the synthesis of iron oxide nanoparticles and thus solve the problem of its storage.

Mill scale generated from the steel industry during the milling process consists of iron oxides (>95%), which are formed under the strong oxidation process of hot-rolled iron products. The waste is recycled into process or valorized, in order to reduce raw material content and to protect the environment [5]. The literature indicates that about 13.5 million tons/year of mill scale are generated in the world [6]. MS represents a peculiar surface of hot rolled steel, with a porous, hard and brittle aspect being a mixture of iron oxides (predominantly FeO and Fe_3_O_4_). The formation of mill scale can also lead to appearance of some corrosion product mixture, such as FeOOH, Fe_2_O_3_, etc. [7]. Usually, FeO is found at the metal surface and Fe_2_O_3_ is in the outer layer [8].

These oxides are formed during the fabrication of steel structures and the wastes can be converted into a valuable secondary material due to its high iron content (up to as high as 93%), low impurities, and stable chemical composition [9,10]. Usually, a reduction process is applied due to the high content of iron, either via hydrogen, by microwave heating, or by applying solution-based techniques [6,11]. MS iron waste has been used a as precursor for pigment preparation [10]. Legodi et al. [12] studied the potential of mill scale iron waste to obtain pigments based on iron oxides. They obtained pigments (from mill scale) under <0.1 mm, with a high surface area. In the case of corrosion product appearance, mill scale has a poor color, exposed hardness, and it is difficult to use as a pigment. Until now, this type of waste obtained from mill scale has not been reported as low-cost nanoadsorbent for waste decontamination. Some countries like Greece consider MS as waste suitable for reuse in the cement industry, steelworks, and the chemical industry [13].

Heavy metals released in water bodies result from various industrial effluents, such as metal plating, electronics, or the power generation industry. Their toxicity and non-biodegradability induce hazardous effects on food chain, with subsequently effects on living organisms [14]. They can be accumulated at low concentrations and can be quickly linked by protein nucleic acids and metabolites, causing the alteration of biological functions [15].

Conventional methods, such as sedimentation, chemical precipitation, solvent extraction, ion exchange, or membrane separation, are well-known for their performances in removing heavy metals from water and wastewater [15]. However, these methods have some disadvantages, such as a large quantity of sludge or solid wastes, chemical reagents, incomplete treatment process, expensive equipment and monitoring requirements. In addition, adsorption has been employed more and more for water treatment, including heavy metal removal. This method is simple, cost-effective, with a simple design and operation [16]. There are a large number of adsorbents that are effective in removing heavy metals, such as carbon-based materials, such as powder or granulated activated carbon [17,18] or carbon nanotubes [19], different synthetic porous materials [20], natural inorganic minerals [21,22], and functionalized polymers [23].

Nanotechnology has provided a new approach to nanomaterial processing [24,25,26]. The application of nanotechnology for water treatment, using nanoadsorbents to remove heavy metals, solves many of the environmental problems, and magnetite and maghemite nanoparticles are very popular. It is well known that for the successful synthesis of Fe_3_O_4_, the use of Fe-containing compound precursors, such as FeCl_2_, FeCl_3_ and FeSO_4_ is mandatory, which also means a high consumption of reagents [27,28,29,30]. Unfortunately, there is not much information in the literature regarding the recycling and reuse of iron compounds.

Along with development and performance of nanotechnology for water decontamination, nanoadsorbent materials have been designed in terms of a high surface area with high reactivity, most of them being used as powders, which are often functionalized or decorated to improve their properties. A disadvantage of these materials is shown in separation and regeneration after heavy metal removal from water. New research has focused on magnetic nanostructured materials to be used as adsorbents for heavy metals due their high reactivity and the ability to use a magnetic field to remove them from water, with the possibility of regeneration [17]. The most well-known magnetic nanoparticles are magnetite (Fe_3_O_4_) and maghemite (γ-Fe_2_O_3_), which have highly magnetic properties [31,32] and are easily separated. There are different methods to synthesize magnetic nanoparticles, such as co-precipitation, hydrothermal synthesis, thermal decomposition, and microemulsion. All of these synthesis methods are effectively controlled and exhibit high productivity [33,34,35]. The capability and reactivity of these magnetic nanomaterials to remove pollutants are different, where pH values are a determining factor for anions’ (i.e., Cr(VI) as CrO_4_^2−^ or Cr_2_O_7_^2−^) and cations’ (i.e., Cu(II), Pb(II), Cd(II) and Ni(II)) selective adsorption [36]. The use of magnetic nanoparticles at specific pH values facilitates the separation and recovery of heavy metals. Regarding an actual approach to the circular economy, one way to capitalize on ferrous waste as a valuable added product is to convert it into low-cost adsorbents with cost-effective and environmental benefits [37]. Taking the economic and environmental considerations into account, the conversion of ferrous wastes into nanoadsorbents for water decontamination has become a challenging task.

Silva et al. [15] reported a method for the production of iron oxide nanoparticles resulting from acid mine drainage. The method is based on the selective precipitation of iron as ferric hydroxide, the dissolution of iron with acid, reduction of Fe^3+^ to Fe^2+^ and changing the ferric/ferrous hydroxides into magnetite [15]. Another method for recovering magnetite and kaolinite from waste iron ore tailing was reported by Giri et al. [2]. They synthesized iron oxide nanoparticles from waste iron ore tailings using acid leaching, followed by precipitation of ferric hydroxide.

Other possible valorization of ferrous wastes is the obtaining of ferrous sulfate heptahydrate from pickling solutions by cooling and evaporating the solution to 5 °C, followed by crystallization of FeSO_4_ at 0 °C [6]. Ferritic nanoparticles could be obtained by microbial oxidation of ferrous sulfate to Fe (III), from pickling solutions, followed by extraction into an organic acid and solvothermal synthesis in the presence of another metal, e.g., Ni or Zn [38,39]. Pickling solutions have been used as precursors for the obtaining of nano-magnetite by ultrasonic treatment-assisted co-precipitation [40].

**Figure 1:**
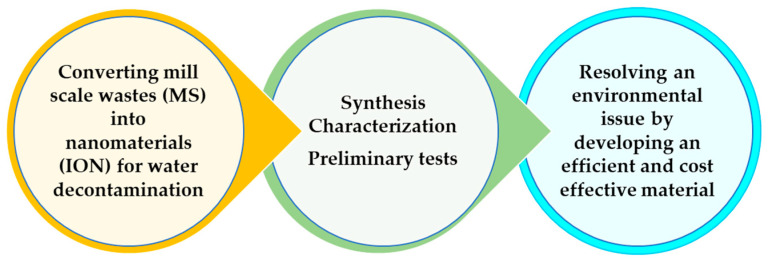
Illustration of the recycling process from mill scale waste to absorbent for the removal of heavy metal ions from waters.

In order to valorize the potential of this material, the main objective of this research is to obtain a magnetic nanoadsorbent for water decontamination, asses the synthesis methods available in the literature and evaluate the performance of magnetic nanostructures as adsorbents for heavy metals (Figure 1).

A magnetic nanoadsorbent was obtained using acidic dissolution, followed by co-precipitation and washing in order to obtain nanosized iron oxides (ION). According to Dehsari et al. [41], iron oxide magnetic nanoparticles obtained by classical chemical synthesis are usually a mixture of two phases: magnetite and maghemite. Because a mixture of magnetite and maghemite was obtained in our work and used as a nanoadsorbent for heavy metals, we will refer to it as ION.

Preliminary adsorption studies have been performed in order to evaluate the capability of ION to remove the heavy metals from aqueous solutions. Adsorption capacity, efficiency and the Langmuir isotherm model are presented in detail.

## 2. Materials and Methods

### 2.1. Steps for MS Converting into ION

**Figure 2:**
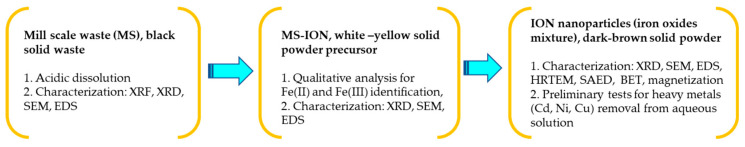
Steps involved in MS conversion into ION, characterization and evaluation as nanoadsorbents.

In order to obtain a valuable product with potential use for wastewater treatment, the following steps (presented in Figure 2) have been developed.

Materials and methods applied for this converting process from waste to a low-cost magnetic adsorbent are described as follows. The mill scale (MS) was purchased from a hot rolling steel factory from Romania. MS classified as waste was used as the raw material in the preparation of ferrous Fe(II) and ferric precursors Fe(III) for the obtaining of ION. The ferrous precursor MS-preparation steps consisted of acidic dissolution in order to obtain a ferric and ferrous precursor as an intermediate product (MS-ION) and a coprecipitation step for obtaining ION as a final product.

#### 2.1.1. Acidic Dissolution Step for MS-ION Preparation as Precursor

In a 600 mL glass beaker, 30 g of MS were treated with 150 mL H_2_SO_4_ conc. (analytical grade). The mixture was heated on a hot plate until dry. The resulting white-yellow muddy solid material with dark solid particles as the intermediate product (MS-ION) was used as starting material for the preparation of magnetite (Fe_3_O_4_) and maghemite (γ-Fe_2_O_3_). This intermediate product was partially soluble in cold water, but with slight heating, the remaining fine dark particles completely dissolved in water and the remaining traces were removed by a decantation. A qualitative test for product solubility in warm water was applied and after dissolution in warm water, 1 mL NaOH was added to give a dark blue/green sediment, which is characteristic of Fe(OH)_2_ in solution [42]. This intermediate solid product (MS-ION), after solubility testing, was cooled and stored in air at 23 °C. A homogenous yellow fine solid product was observed after 5 days of exposure. In addition, its solubility in warm water was maintained, and after 1 mL NaOH was added, a rust-colored sediment was observed. This behavior is also characteristic of the presence of Fe(OH)_3_ [42]. These qualitative tests were further validated by quantitative analyses for the Fe(II) and Fe(III) species.

#### 2.1.2. Coprecipitation Method for ION Preparation

Iron oxides as IONs were obtained from 40 g MS-ION dissolved in 250 mL of distilled water. After filtration in a Buchner funnel, the liquid solution was stirred at 200 rpm with 60 mL NaOH 40% for 30 min. The pH value was raised at 12 and a dark brown solid product resulted, i.e., ION. The mixture was aged at room temperature for 1 day, then filtered and washed with ultrapure water until pH 7. ION was dried at 60 °C and prepared for characterization and preliminary adsorption tests as nanoadsorbent material. The balance of materials was calculated and it was found that about 250 g of ION (Fe_3_O_4_, γ-Fe_2_O_3_) could be obtained from 1 kg of MS precursor.

### 2.2. Characterization and Composition of MS, MS-ION and ION

The composition of mill scale (MS) obtained from the hot rolled steel line was established with a wavelength dispersive X-ray fluorescence (XRF) spectrometer (AXS S8 Tiger, Bruker, Billerica, MA, USA). Previously, 10 g of MS were ground to a manageable fine grain size of about 4 µm using a Retsch PM 100 type ball mill, containing WC balls. The MS powder was then mixed with boric acid as a binder and pressed at 40 tons using a Pellet-Press machine Retsch PP 40, Retsch GmbH, Düsseldorf, Germany.

To obtain a comprehensive understanding of the MS waste from compositional and structural point of view, and how it could be used as a precursor for ION synthesis, several characterizations were carried out, including MS-ION characterization. X-ray diffraction (XRD) analysis was performed to investigate the phases, structure and purity using a PANalytical X’Pert PRO MPD spectrometer with a Cu anode. In order to determine the crystallite size of the nanoparticles, the Debye-Scherer equation (Equation (1)) was used [43]:

(1) $$D=\frac{Kx\lambda_{Cu-K\alpha }}{\cos\theta x\;FWHM}$$

where *D* is the crystallite size; *K* = 0.89 is the dimensionless shape factor, *λ_Cu-Kα_* is the radiation wavelength for the X-ray tube; *FWHM* is the width at half of the maximum diffraction (in radians), and *θ* is the diffraction angle.

The morphology and size of the MS, MS-ION, and ION were investigated via scanning electron microscopy (SEM, Quanta Inspect F type), with field emission gun, 1.2 nm resolution, coupled with energy dispersive spectra (EDX). The size was validated by high resolution transmission electron microscopy (HRTEM) (TECNAI F30 G2 with linear resolution of 1 Å and point resolution of 1.4 Å) with selected area electron diffraction (SAED) capability. Additional, quantitative analysis of the soluble MS-ION precursor was achieved. Thus, 1.95 g MS-ION were dissolved in 100 mL warm water. The water solution was then analyzed by spectrophotometry to determine the Fe (III) amount in the solution. The ortho-phenanthroline method was applied to analyze Fe (III) content, which was based on a red-orange compound formation. The compound was measured at λ = 510 nm using a molecular absorption spectrometer (Cintra 220 GBC) with a spectral domain between 190 and 1000 nm. These results obtained for Fe (III) were compared to XRF analysis and atomic absorption spectrometry (AAS) to determine the total dissolved Fe (Fe II + Fe III) content. Thus, an atomic absorption spectrometer (ContraAA800D Analytik Jena) equipped with a Xe lamp, flame, and double beam optical system, with a spectral range between 185 and 900 nm, was used.

### 2.3. Magnetic Properties and BET Surface Analysis

Magnetic properties were studied using a vibrating sample magnetometer (VSM 880—ADE Technologies, Westwood, MA, USA) at room temperature. Additionally, the specific surface area used for identification of pore volume and the surface characteristics were measured by BET (Brunauer, Emmett and Teller) using a NOVA 2200e-Quantachrome Analyzer, Quantachrome, Boynton Beach, FL, USA in order to establish heavy metal adsorption mechanism.

### 2.4. Adsorption Tests

**Figure 3:**
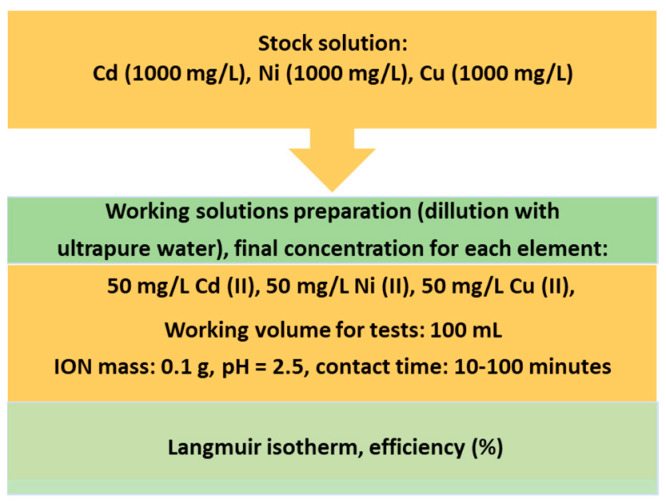
Schematic procedure for the preparation of the ternary heavy metals (Cd, Ni, Cu) aqueous system.

Preliminary adsorption tests were developed in order to establish future working protocols for mechanism, kinetic and thermodynamic studies for ION applications as nanoadsorbents. All the reagents used in the adsorption test were of chemical grade, purchased from Merck, with quality certificates. Adsorption tests were applied to validate the heavy metal removal efficiency using ION as nanoadsorbent in a ternary-heavy metal system aqueous solution. In this regard, a multicomponent solution of 1000 mg/L of each element (Cd, Ni, Cu) was diluted in order to obtain a working solution of 50 mg/L of each element. Thus, 100 mL of solution was put into contact with 0.1 g ION, at a pH 2.5, with a contact time of between 10 and 100 min. The aqueous solutions were initially stirred at 200 rpm to allow for the adsorption of heavy metals ions onto the ION particles. After stirring for 10 min, 1 mL aliquot of supernatant was sampled with the help of an external magnet. Then, stirring was continued and sampling of the solution was repeated every 10 min. All the experiments were performed twice for data analysis. The ION nanoadsorbent was extracted and recovered from the system using an external magnet. Schematically, the steps for the ternary system preparation are presented in Figure 3.

Testing of the ION adsorption capacity was validated by heavy metals concentration analysis, before (*C*_0_) and after (*C*_t_) contact with ION. The concentrations were measured using an atomic adsorption spectrometer (ContraAA800D Analytik Jena, Jena, Germany), with a Xe lamp, flame and double beam optical system, and a spectral range between 185 and 900 nm. Specific wavelengths for the heavy metal ions investigated in this study were established at: 228.8 nm, 232 nm, and 324.7 nm for Cd, Ni, and Cu, respectively.

The removal efficiency (%) that indicated the quantity adsorbed onto ION surface as mass balance between concentrations values was calculated using Equation (2):

(2) $$\eta \;(\%)=\frac{\left(C_{0}-C_{t}\right)}{C_{0}}\times 100$$

where *C*_0_ and *C_t_* are the initial and final concentrations of heavy metals (mg/L).

In order to evaluate possible forces of attraction between the solute molecules of heavy metals from the solution onto the solid ION surface, Langmuir isotherm models were applied as a preliminary investigation of the adsorption mechanism.

The Langmuir equation describes heavy metal behavior at a constant temperature (25 °C) on the adsorption capacity, based on Equation (3) [44]:

(3) $$\frac{C_{e}}{q_{e}}=\frac{1}{Q_{max}\times b}+\frac{C_{e}}{Q_{max}}$$

where *C_e_* is the equilibrium concentration of the adsorbate (mg/L); *q_e_* is the adsorbed metal amount (mg/g), *Q_max_* is the maximum adsorption capacity (mg/g), and *b* is the Langmuir constant, L/mg.

The Langmuir equilibrium constant, *K_L_*, can be expressed as: *K_L_* = *Q_max_ x b*. In addition, separation factor *R_L_*, defined by Equation (4) [45,46], was used for evaluating the nature of the adsorption process.

(4) $$R_{L}=\frac{1}{\left(1+b\times C_{0}\right)}$$

A favorable adsorption process has *R_L_* values between 0 and 1, while for *R_L_* > 1 the adsorption process is unfavorable; for *R_L_* = 1 a linear adsorption take place and *R_L_* = 0 represents an irreversible adsorption process [47].

The adsorbed metal amount at equilibrium was calculated by Equation (5):

(5) $$q_{e}=\frac{\left(C_{0}-C_{e}\right)}{V}\times m$$

where *C*_0_ is the initial concentration, mg/L; *C_e_* is the equilibrium concentration, mg/L; *V* is the volume of the solution, L; *m* is the adsorbent quantity, g. Based on the Langmuir model results, future experiments will be developed for heterogeneous surface study in accordance with the Freundlich isotherm.

## 3. Results and Discussion

### 3.1. Characterization and Composition of MS

The XRF analysis of MS was achieved using pellets obtained after grinding and compressing the MS powder. The elemental composition is shown in Table 1. The average content of Fe was about 77%, as a major element of MS, followed by O, Ca, Si, and Mn. The values are an average of five replicates of MS.

**Figure 4:**
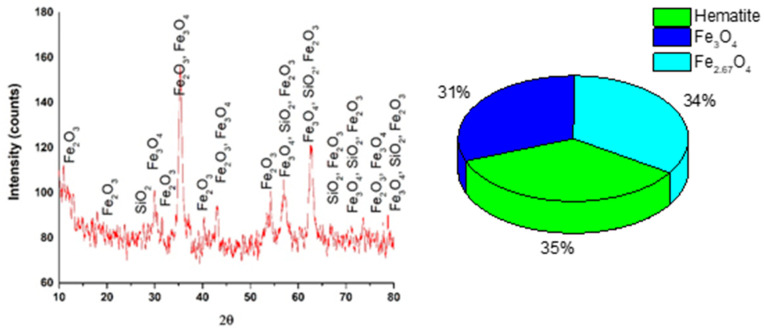
X-ray diffraction of the MS powder and percentage of identified phases (inset).

XRD analysis of MS indicated that morphological phases of this material are represented by a mixture of iron oxides as can be observed in Figure 4.

**Figure 5:**
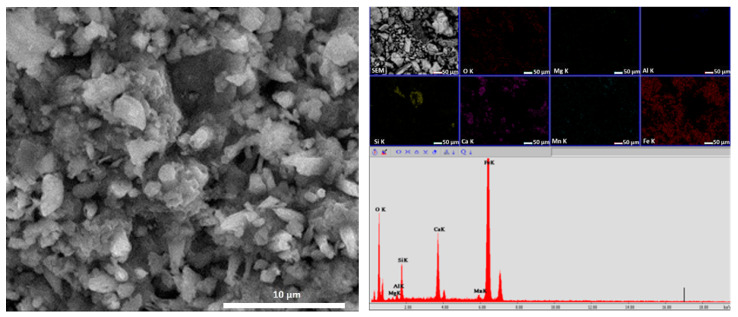
SEM image of the MS powder at 10,000× and EDX associated.

X-ray diffraction spectrum of the MS indicated as first phase Fe_2_O_3_ (35%) as hematite, followed by Fe_2.67_O_3_ (34%) and Fe_3_O_4_ (31%). These oxides were identified in accordance with the ICSD data sheet. The crystallite size was calculated using Equation (1) and was about 135 nm. In addition, the structure, aspect and size of MS particles were identified by SEM coupled with EDX, as can be seen in Figure 5.

From Figure 5, the agglomeration of MS particles can be seen, together with different shapes, and of the analyzed MS. Using the EDX technique, spectra indicated the presence of Fe, Ca, Si, Mn, Al and Mg; this information being in accordance with the XRF analysis. Mapping information revealed a homogenous distribution of Fe and a concentration of Si and Ca, other mentioned elements were in the mass of the sample.

### 3.2. Characterization and Composition of MS-ION

**Figure 6:**
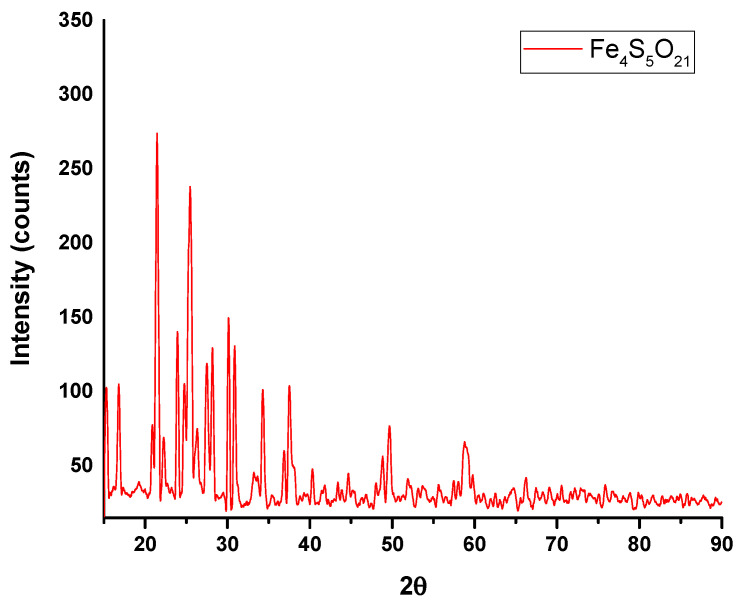
X-ray diffraction of the MS-ION powder.

After acidic dissolution and evaporation/drying steps, a white-yellow solid material resulted, which was used as a precursor (ION-MS). XRD spectra (Figure 6) indicated that acidification converted MS into a complex major phase as Fe_4_S_5_O_21_, according to the ICSD data sheet. This compound is characteristic of MS-ION as an intermediate product for ION preparation. The crystallite size was calculated using Equation (1) and was about 75 nm.

**Figure 7:**
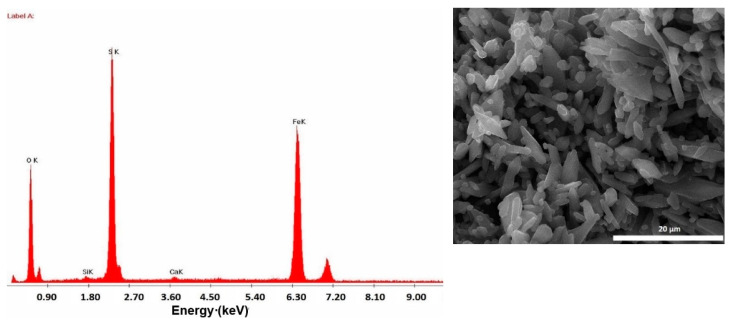
EDX results obtained on the SEM image (inlet) of the MS-ION precursor.

Figure 7 presents the SEM-EDX observations performed on the MS-ION material. Particles are agglomerated, with a well-defined elongated aspect. In addition to these shapes, there are small and rounded particles of about 200 nm. This size is in accordance with the small crystallite size of the particles generated by the Debye-Scherrer equation; the SEM observations indicate the agglomeration of these crystallites. The round shape is characteristic of powders obtained by a ball grinding process.

According to the EDX image, the major elements are Fe, S and O, which compares well with the XRD spectra. In order to establish Fe speciation from the MS-ION precursor, a traceability analysis method was developed, which combined XRF and AAS techniques. The results obtained are presented in Table 2. For 1.95 g MS dissolved in warm water, an average percentage of 77% Fe could be estimated. This quantity is in accordance with results indicated in Table 1 for MS analyzed by XRF. This indicated that the initial Fe content was maintained and a ratio of 1:4 (*w*/*w*) of Fe(II): Fe(III) was available as a mixture in the MS-ION precursor.

The total Fe concentration (sum of Fe(II) and Fe(III)) analyzed using the AAS technique corroborated with the XRF analysis and spectrophotometry technique and indicated that about 20% represents Fe(II). With this information, according to the general chemical reaction of iron oxides preparation, the specific molar ratio is settled at 1:2 for FeCl_2_:FeCl_3_ [46]. We worked within an excess of Fe(III) that can be explained by the original composition of the used waste and can be observed by XRD and EDX analyses for MS.

### 3.3. Characterization and Composition of ION

**Figure 8:**
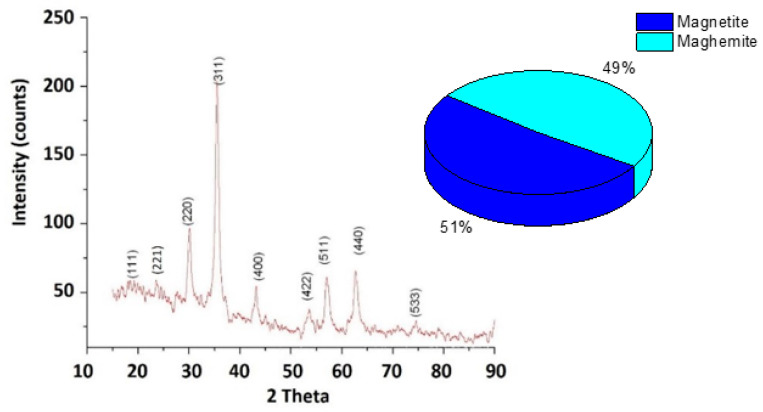
XRD results of the IONs powder.

ION-MS was treated using the coprecipitation method [48,49]. XRD spectra presented in Figure 8 for ION show a cubic crystal system according to the ICSD datasheet. The majority phase is represented by a mixture of Fe_3_O_4_ and γFe_2_O_3_ compounds. The overall XRD pattern associated with the percentage of compounds indicated that the ION sample contains a mixture of amorphous and crystalline phases.

**Figure 9:**
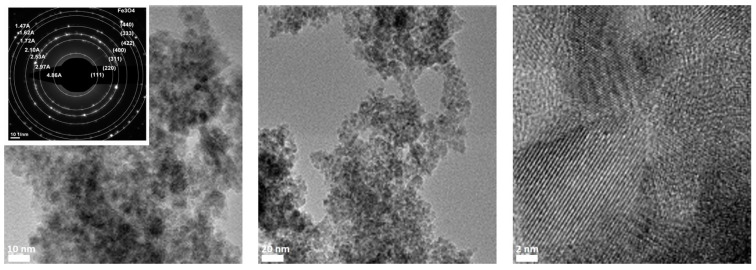
TEM images at different magnifications of ION. The inlet shows the SAED diffraction pattern.

The width of the peaks suggests that the crystals are of nanometer dimensions (Figure 8). Using the Debye-Scherrer formula, a crystallite particle size of 9.3 nm was calculated. The nanometric size of ION was confirmed by TEM coupled with the scanning area electron diffraction (SAED) technique, as shown in Figure 9.

**Figure 10:**
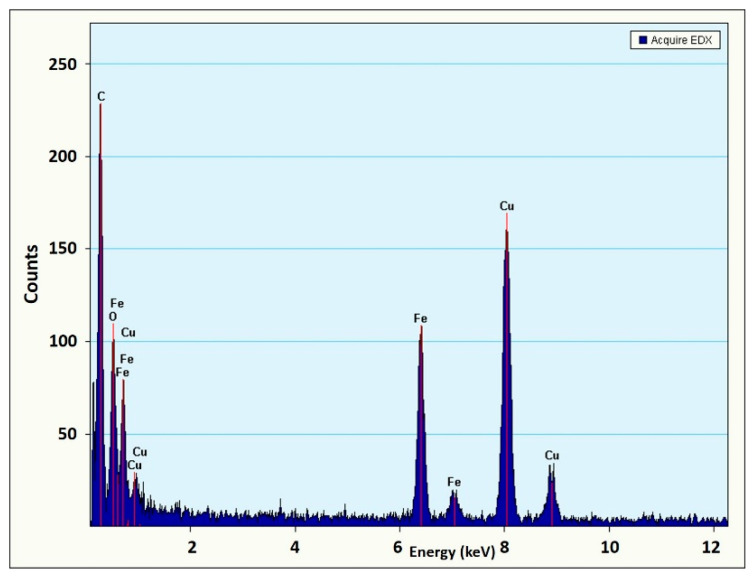
EDX image of the ION sample corresponding to the TEM image presented in Figure 9.

The HRTEM images (Figure 9) show the crystalline nature of the ION sample. Diffraction analysis (SAED) confirmed the presence of Fe_3_O_4_, as shown in the inlet in Figure 9. The SAED image shows clear rings with interplanar spacings associated with the corresponding (h k l) Miller indices for Fe_3_O_4_. Additionally, X-ray microanalysis in dispersive energy (EDX) confirms that the sample contains Fe and O, with a Cu presence being associated with the Cu grid used for sample analysis (Figure 10).

**Figure 11:**
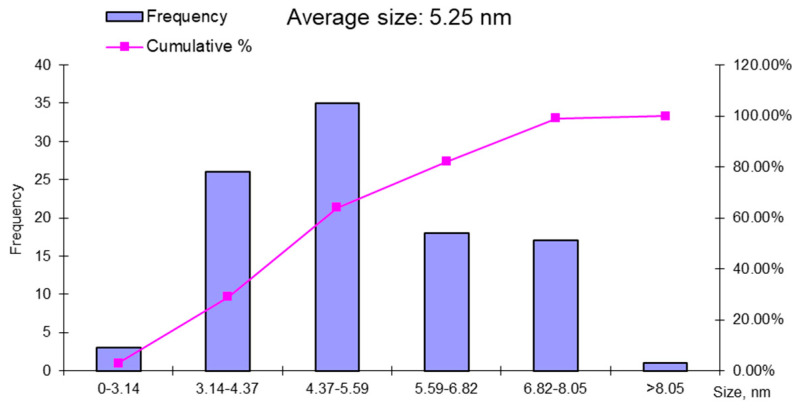
Histogram representing the distribution of the magnetite nanoparticles, as synthesized from the mill scale waste.

The average particle size of the iron nanoparticles was 5.25 ± 19 nm, with an average of 100 nanoparticles in the TEM image. The histogram presented in Figure 11 shows that the most nanoparticles were around 6 nm, but not below 3 nm. The low frequency of nanoparticles of 8.05 nm confirms that most nanoparticles had dimensions around the average of 5.25 nm.

### 3.4. Magnetic and Surface Properties Investigations

The magnetic properties of ION are the key factor in the use of nanomaterials as sorbents for water and wastewater treatment of heavy metal ions [50]. Adding magnetic properties to nanoparticles allows for nanoadsorbents to be magnetically separated. Our research group developed a solenoid column for the removal of heavy metal ions using magnetic nanoparticles [51]. Therefore, the magnetic properties of ION nano-powders are as important as their adsorption properties for water treatment applications.

**Figure 12:**
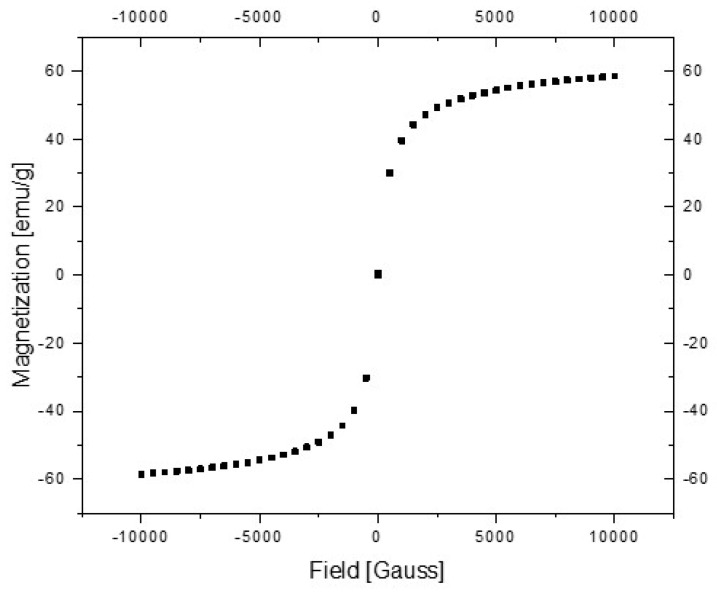
Magnetization curves (M) vs. magnetic field strength (H).

Figure 12 presents the magnetization curve function of the magnetic field strength, H, measured in the range of H∈[−10,000,+10,000] Gauss. Because the hysteresis curve area is null, the ION nanoparticles exhibit superparamagnetic behavior at room temperature. Additionally, the shape of the magnetization curve indicates the nanomaterial characteristics of the powder.

According to Ilyushenkov et al. [52], the saturation magnetization of “bulk” ION is about 70 emu/g. Since the saturation magnetization decreases with the decrease in particle size, the nanosized dimension of the particles can be accounted for and the saturation magnetization of 55 emu/g obtained for ION nanoparticles. The results indicated a mixture between Fe_3_O_4_ and γFe_2_O_3_ with the possibility of having nanoparticles with a magnetite core covered by a maghemite layer.

**Figure 13:**
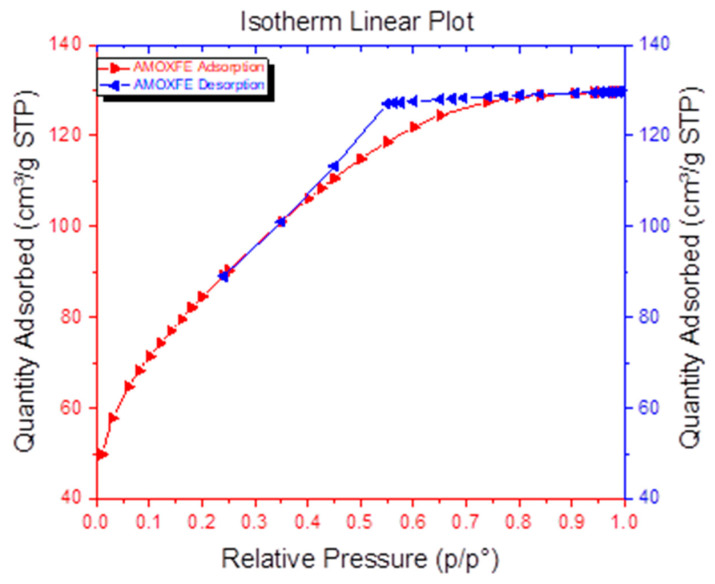
Specific surface area of the manometric magnetite.

Specific surface assays performed by BET analysis (Figure 13) were used to calculate the specific surface for monolayer adsorption. The following assumptions were made: adsorption occurs on a homogeneous surface and there are no interactions between molecules. The isotherm obtained for the ION sample suggests a multilayer adsorption.

The results obtained from the adsorption isotherm have shown that the ION sample is a mesoporous powder with an average pore size of <5 nm and a pore specific surface area of 146.559 m^2^/g. The sample is characterized by a large specific surface area of 301.54 m^2^/g and an average particle size of 6.6 nm. Compared to other data from the literature, the ION powder shows a good adsorption capacity of about 8 nm for which the BET surface is 95.3 m^2^/g [53]. Future investigations will be developed. In accordance with other results obtained with the nanostructured iron oxides used for the removal of heavy metals from wastewaters [49,51], the use of MS for ION preparation led to a porous and active nano-adsorbent with promising results for heavy metal removal.

### 3.5. Adsorption Tests

The research regarding the adsorption of heavy metals was conducted by adding 0.1 g of ION as an adsorbent for heavy metals from a ternary solution system according to the procedure presented in Figure 3. To study the kinetics of the adsorption process, the ternary system solution was kept in contact for different periods of time (between 10 and 100 min), starting at pH 2.5 as the initial pH value of the aqueous solution. The separation of the ION adsorbent was performed using a magnet and the sampled supernatant was analyzed from 10 to 100 min by AAS in order to establish the final concentration of heavy metal, after contact with the ION adsorbent.

**Figure 14:**
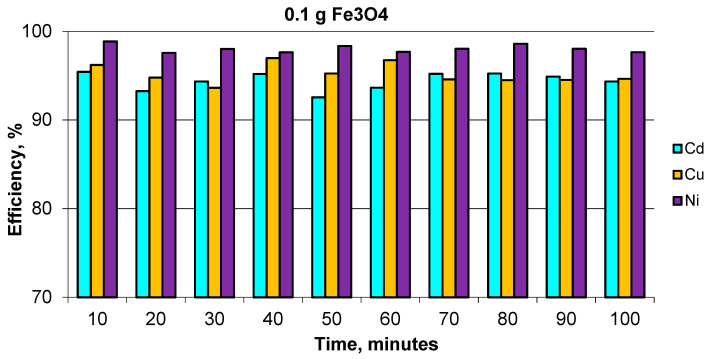
Removal efficiency of Cd, Ni and Cu for ION.

The removal efficiency of Cd, Cu, and Ni of magnetite nanoparticles can be observed in Figure 14, which shows good yields for the removal of heavy metals, with a retention efficiency of more than 95% for Ni and over 90% for Cu and Cd. The maximum efficiency was reached after 10 min and was maintained approximately the same under all of the times tested. The difference in removal efficiency for Ni(II), Cu(II) and Cd(II) could be influenced by a number of factors, such as hydration radii and solubility of the cations. Thus, the hydration radii values of the cations are rHNi^2+^ = 4.04 Å, rHCu^2+^ = 4.19 Å and rHCd^2+^ = 4.26 Å [54]. The smallest cations should ideally be adsorbed faster and in larger quantities compared to larger cations, since the smaller cations can pass through the micropores and channels of ION that was used as an adsorbent.

**Figure 15:**
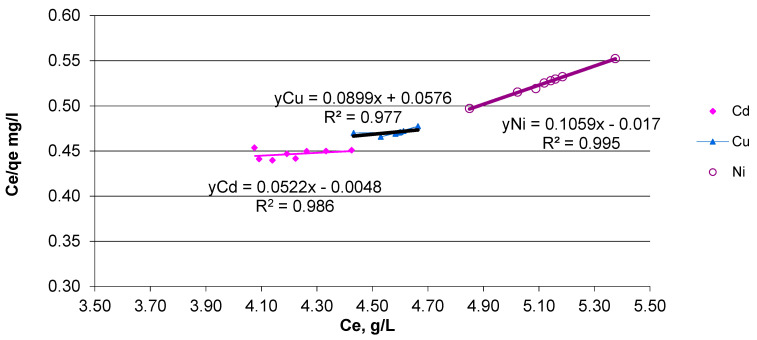
Linearized Langmuir model for Cd, Ni and Cu adsorbed onto ION.

A decrease in the heavy metal concentration was observed after the first 10 min in the order of: Cd < Cu < Ni. Increasing the contact time to 100 min did not improve the metal adsorption onto magnetite nanoparticles. Based on the efficiency results, the maximum adsorption capacity (q_e_) was calculated in accordance with the concentration values at equilibrium (C_e_) for each heavy metal ion. In order to validate these data, the Langmuir isotherm model was applied for ternary system solutions. The collected data are in accordance with the Langmuir model calculated using Equation (3), for monolayer adsorption, as shown in Figure 15.

According to the Langmuir isotherm, the q_e_ values are: 9.44 mg/g (Ni), 11.12 mg/g (Cu) and 19.15 mg/g (Cd). The correlation factor (R^2^) indicates a good fitting of the results for Ni (0.99), followed by Cd (0.98) and Cu (0.97). Further models can be explored in the future to evaluate the adsorption process. The preliminary results indicated that the heavy metal ions were adsorbed in accordance with Langmuir relation, where the solid (adsorbent) surface exposes a specific number of sites that, once occupied, permit no further adsorption to occur [55]. In addition, separation factor R_L_, calculated with Equation (4) indicates a favorable process for ternary solution systems. Thus, because the R_L_ values are between 0 and 1, we can assume that the process describes a favorable adsorption process.

## 4. Conclusions

Iron oxide nanoparticles, noted here as ION, have been synthesized from mill scale waste and thoroughly characterized to explore their use as nanoadsorbent for heavy metals from aqueous solutions. The MS converted into iron oxide nanopowders were characterized by X-ray, SEM, TEM, EDX, BET, and magnetic properties. The iron oxides nanoparticles (as mixture of Fe_3_O_4_ and γ-Fe_2_O_3_) obtained from the MS powder were about 6 nm in size and presented superparamagnetic behavior and a large specific surface area. All these properties of the magnetite nanopowder synthesized from the MS powder highlighted their potential for use as nanoadsorbents for heavy metal removal from wastewaters. Preliminary studies regarding heavy metals ions (Ni, Cu, Cd) from ternary aqueous solution systems were performed with good removal efficiency, i.e., over 90% after 10 min, which was maintained for about 100 min. Additionally, the data are well fitted with the Langmuir isotherm, thus futures studies will be developed in order to establish the adsorption mechanisms and chemistry surface of ION as a product resulting from industrial waste (MS). Additionally, the technology proposed in this paper, which can convert 1 kg of mill scale powder to 250 g of nanomagnetic iron oxides (Fe_3_O_4_, γ-Fe_2_O_3_) points to an efficient recycling and reuse technology of mill scale, a waste from steel production.

## Figures and Tables

**Table 1:** Chemical composition of mill scale waste.

Element	Average Content, wt.%
Fe	77.06 ± 0.04
O	13.35 ± 0.15
Ca	4.50 ± 0.26
Si	1.90 ± 0.88
Mn	0.83 ± 0.41
Na	0.71 ± 5.72
Al	0.64 ± 1.87
Mg	0.22 ± 4.40
P	0.18 ± 2.51
Cr	0.04 ± 2.26
Ti	0.03 ± 4.01

**Table 2:** Fe concentration from the MS-ION precursor.

Fe Concentration, mg/L	Analysis Method
Fe tot = 242.93	AAS (liquid)
Fe tot = 247.92	XRF (liquid)
Fe(II) = 50.80	Spectrophotometry (liquid)

## Data Availability

The data presented in this study are available on request from the corresponding author.
